# Editorial: Harnessing biomechanotransduction to influence cell fate

**DOI:** 10.3389/fbioe.2023.1357961

**Published:** 2024-01-05

**Authors:** Peng-Yuan Wang, Hosein Shahsaverani, Hsien-Yeh Chen, Matthew John Dalby

**Affiliations:** ^1^ Oujiang Laboratory, Key Laboratory of Alzheimer’s Disease of Zhejiang Province, Institute of Aging, Wenzhou Medical University, Wenzhou, China; ^2^ Department of Cell and Molecular Biology, Faculty of Life Science and Biotechnology, Shahid Beheshti University, Tehran, Iran; ^3^ Department of Chemical Engineering, National Taiwan University, Taipei, Taiwan; ^4^ School of Molecular Biosciences, University of Glasgow, Glasgow, United Kingdom

**Keywords:** stem cells, mechanotransduction, epigenetic state, materiobiology, mechanobiology, biophysical cues, cell reprogramming, surface topography

Cells, the fundamental units of life, are not passive entities solely following genetic instructions; they actively respond to the physical forces in their microenvironment. Recent breakthroughs in cell biology emphasize the role of biophysical cues in directing cell fate, revealing a fascinating interplay between the physical surroundings and cellular destiny. This editorial explores the emerging field of biophysical cues and its implications for cell fate regulation.

Traditionally, cellular biology has centered on molecular signals–the intricate web of genetic instructions and chemical messengers guiding cells along developmental paths. However, researchers now recognize that a cell’s physical surroundings, encompassing mechanical forces, substrate stiffness, and topographical features, play equally influential roles in sculpting cellular destiny.

Biophysical cues possess the remarkable ability to mimic a cell’s natural environment. Cells, residing in tissues with diverse mechanical properties, finely tune their behaviors to respond to these variations. Mechanotransduction, the interplay between cells and their surroundings, is exemplified by substrate stiffness mimicking the extracellular matrix (ECM). For example, stem cells on brain-tissue-mimicking substrates tend to differentiate into neural lineages. On the other hand, mechanical memory is also crucial; cells may remember microenvironmental conditions, participating in fate regulation afterward by altering epigenetic states.

Understanding how the physical microenvironment influences stem cell differentiation can revolutionize regenerative medicine. Scientists aim to orchestrate stem cell transformation into specific cell types by providing the right biophysical cues, offering unprecedented control over regeneration. The intriguing role of mechanotransduction in stem cell biology highlights stem cells’ sophisticated mechanosensing machinery. This machinery interprets mechanical cues in the microenvironment, allowing stem cells to make crucial fate decisions. For example, molecular clutch regulation of cytoskeletal tension in response to substrate elasticity, can drive osteogenic factors into the nucleus and lock in bone-related differentiation programs. During embryonic development, mechanical forces influence cell differentiation into specialized cell types. Also, tissues undergo dynamic mechanical changes, and cells respond by adapting their fate accordingly. Disruptions in these mechanical cues can lead to developmental abnormalities, emphasizing mechanotransduction’s integral role in life’s earliest stages.

Mechanotransduction’s implications extend beyond stem cells. Researchers leverage this knowledge to design biomaterials guiding cell fate actively, envisioning scenarios where biomaterial scaffolds, through their mechanical properties, coax cells to regenerate damaged heart tissue or repair injured spinal cords. Microscale and nanoscale topographical features add another fascinating dimension to biophysical regulation. Cells sense and respond to the physical architecture of their surroundings, influencing morphology, gene expression, and differentiation. Stretching, compression, and shear stress, which cells experience *in vivo*, play pivotal roles in tissue development and homeostasis. The mechanotransduction machinery within cells converts these physical signals into biochemical responses, steering cellular behavior. Unraveling mechanotransduction intricacies holds promise for interventions in conditions where mechanical cues are altered, such as cardiovascular diseases and musculoskeletal disorders.

Moreover, mechanotransduction plays a crucial role in the cell reprogramming of differentiated cells into a pluripotent state, a process vital for regenerative medicine and disease modeling. Cell reprogramming, demonstrated by induced pluripotent stem cells (iPSCs), involves coaxing mature cells to revert to a pluripotent state. The mechanical cues within the cellular microenvironment during cell reprogramming significantly influence the process’s efficiency and fidelity. At the same time, transdifferentiation, where cells transform from one specialized identity to another without reverting to a stem cell state, challenges conventional boundaries of cellular fate. While the focus historically has been on genetic and molecular cues guiding transdifferentiation, recent revelations highlight the integral role played by mechanical forces in this transformative process.

Understanding mechanotransduction holds significant implications for disease, especially in conditions like cancer where aberrant mechanosensing contributes to progression and metastasis. Cancer cells often exhibit altered responses to mechanical cues, allowing them to evade normal regulatory mechanisms maintaining tissue integrity. Unraveling mechanotransduction pathways in cancer cells opens avenues for therapeutic interventions disrupting aberrant signaling.

In this Research Topic, 2 original research and 3 review articles were collected. Hu et al. review the JAK/STAT pathway’s multifaceted roles, emphasizing its importance in mechanotransduction and therapeutic strategies. Chen et al. summarize the objective of bioimplant engineering to develop biologically compatible materials for orthopedic applications, addressing nanotechnology’s potential and challenges in orthopedics. Wang et al. comprehensively discuss intracerebral hemorrhage (ICH) pathology, active drugs, and nanotechnological applications for efficient ICH therapy. Ahmadian et al. introduce a novel 3D scaffold using decellularized tomato hairy leaves to mimic human hepatocellular carcinoma (HCC) microenvironments for drug testing. Hamrangsekachaee et al. investigate GCX-regulated mechanotransduction using a collagen-derived gelatin matrix, providing insights into endothelial functions under different conditions.

In conclusion, integrating biophysical cues into our understanding of cell fate regulation represents a paradigm shift in cellular biology. Cells are dynamic entities profoundly influenced by the physical world around them, not just biochemical entities responding to molecular signals. This evolving field opens avenues for groundbreaking therapies, providing hope for regenerative medicine, tissue engineering, and a deeper understanding of developmental processes.

